# Obstetrics and Gynæcology

**Published:** 1877-02

**Authors:** 


### III. Obstetrics and Gynæcology.

1. *Fatal Hæmorrhage in the Case of a Pregnant Woman affected with Varix.* POLAILLON. (*Gaz. Obstetric*, October 5, 1876.)

A young woman, twenty to twenty-five years old, by occupation a laundress, was in the habit of walking every morning from Gentilly to St. Jacques, though affected with varicose veins of the legs. One morning, after her usual walk, she noticed blood escaping from a wound in the leg. The hæmorrhage becoming abundant, she was placed on a litter and carried to the Maternity Hospital, but died before arriving in the ward.

A living child was extracted by the Cæsarean operation, but died a few instants after. A small circular wound was discovered over the internal malleolus of the mother, whence the hæmorrhage had proceeded. The garters surrounding the limb above the knee had produced there a marked constriction.

Instances of this sort are rare, but yet have been noted. P. had observed one case a few years before, where death resulted in the case of a woman who injured a varix of the leg upon the step of a stair which she was ascending.

Cazeaux cites an analogous case of hæmorrhage from the labia majora, which resulted in death. A varicose vein of the vulva had been ruptured, and it was believed that the hæmorrhage came from the uterus. In the discussion of this case before the Société de Médecine, it was thought that the albuminuria of pregnancy might have some etiological significance in the explanation of the fatal issue, as well as the myo-carditis, which is relatively frequent in the puerperal state.

2. *Active Hæmorrhage produced by Coitus.* CRANDALL. (*Med. and Surg. Reporter*, October 28, 1876.)

The patient was thirty-five years old, and had been married three weeks. She had been flowing profusely since the first coitus, and was getting very weak. An examination (with speculum) discovered the blood to come from the inside of the uterus. This organ in position, shape, size and appearance was normal. It was learned that she had just ceased menstruating when this first sexual act occurred, and it was inferred to be a case of "intra-uterine hæmorrhage, produced by coition taking place soon after the menstrual flow had ceased, and while the ovaries and uterus were in a highly congested state."

Ergot and gallic acid in liberal doses failing to at all lessen the flow, the tampon was resorted to, when, this failing, the unguentum ferri was applied to the intra-uterine walls with Burnes' uterine ointment positor. This stopped the flowing, and the recovery was only retarded by a slight fever.

The author mentions another case of a similar sort, occurring several years previously, where a woman the next day after her marriage was attacked with a hæmorrhage while sitting at dinner, the flow being so profuse as to come "near proving fatal."

[A case has come to our knowledge recently where a first coitus in a young girl caused such a hæmorrhage as to frighten her paramour, and cause him to run to a doctor for some styptic medicine. From his description it must have been some solution of iron. On being applied to the orifice of the vagina, it quickly checked the bleeding, and we infer hence that the accident was a sudden rupture of the hymen. The young woman — an unusually vigorous person — was so much debilitated that she was obliged to keep her bed for several days.—EDS.]

